# Management of the Unexpected Difficult Airway in Neonatal Resuscitation

**DOI:** 10.3389/fped.2021.699159

**Published:** 2021-10-28

**Authors:** Gazmend Berisha, Anne Marthe Boldingh, Elin Wahl Blakstad, Arild Erlend Rønnestad, Anne Lee Solevåg

**Affiliations:** ^1^The Department of Paediatric and Adolescent Medicine, Akershus University Hospital, Lørenskog, Norway; ^2^Institute of Clinical Medicine, Faculty of Medicine, University of Oslo, Oslo, Norway; ^3^The Department of Paediatric and Adolescent Medicine, Oslo University Hospital, Oslo, Norway

**Keywords:** newborn, resuscitation, endotracheal intubation, difficult airway, algorithm

## Abstract

A “difficult airway situation” arises whenever face mask ventilation, laryngoscopy, endotracheal intubation, or use of supraglottic device fail to secure ventilation. As bradycardia and cardiac arrest in the neonate are usually of respiratory origin, neonatal airway management remains a critical factor. Despite this, a well-defined in-house approach to the neonatal difficult airway is often lacking. While a recent guideline from the British Pediatric Society exists, and the Scottish NHS and Advanced Resuscitation of the Newborn Infant (ARNI) airway management algorithm was recently revised, there is no Norwegian national guideline for managing the unanticipated difficult airway in the delivery room (DR) and neonatal intensive care unit (NICU). Experience from anesthesiology is that a “difficult airway algorithm,” advance planning and routine practicing, prepares the resuscitation team to respond adequately to the technical and non-technical stress of a difficult airway situation. We learned from observing current approaches to advanced airway management in DR resuscitations in a university hospital and make recommendations on how the neonatal difficult airway may be managed through technical and non-technical approaches. Our recommendations mainly pertain to DR resuscitations but may be transferred to the NICU environment.

## Context

Akershus University Hospital (AUH) has ~5.000 deliveries per year and the level 2–3 NICU provides care for newborn infants from 26 weeks gestation. A pediatric resident and a general pediatrician are responsible for initial resuscitation and stabilization of newborns in the evening and nighttime with an on-call neonatologist at home with a 30-min response time. The neonatal resuscitation team is called for when a baby is in unexpectedly poor condition at birth, and consists of a pediatric resident, a consultant neonatologist (or pediatrician) and a nurse from the NICU. At cesarean deliveries, more rarely in vaginal deliveries, one or more anesthesiologists is/are present for the care of the mother and will offer assistance if the neonate is depressed and requires resuscitation. The regional and national referral hospital that cares for most of the extremely pre-term infants in the region, is located only 21 km away.

## Introduction

Roughly 5% of newborns need respiratory support such as positive pressure ventilation (PPV) to successfully overcome the phase from fetal to extrauterine life. Of these 3–10% may not respond to mask ventilation leading to an attempt to intubate (1–3). Airway management is a core skill in neonatology and anesthesiology. Endotracheal intubation (ETI) of neonates in delivery rooms (DR) and neonatal intensive care units (NICU) is a procedure associated with a risk of complications and airway injury. The DR and NICU serve different purposes in the stabilization of organ systems, development, and growth. DR medical care emphasizes support of the immediate period after birth where resuscitation including advanced airway management is often anticipated. NICU medical care more often focuses on maintaining stabilized organ systems and providing nutrition for development and growth (4, 5). The incidences of acute resuscitative NICU intubations varies between units but are often less expected (4). Analysis of ETIs performed in the DR and NICU shows that significant differences in patient, provider, practice characteristics, and in the use of airway adjuncts exist (6).

### Anatomical and Functional Differences Between Neonates and Older Children and Adults

Knowledge of neonatal airway anatomy is important during ETI to avoid pharyngeal obstruction, and to accurately assess the larynx. In airway management, the key anatomical structures are located from the lips and nostrils to the carina. Distinct features of the neonatal airway, also seen at laryngoscopy, compared to older children/adults include (7):

- Neonates are preferential nasal breathers and may have difficulty breathing through the mouth to sustain respiration when the nostrils are obstructed.
- The tongue is relatively large.
- The epiglottis is larger, longer, less flexible, and narrower.
- The larynx lies higher and more anterior to the cervical vertebrae reaching the ‘adult' position by around 6 years of age.
- The larynx is funnel-shaped and the subglottis (the outlet of the cricoid ring) is the narrowest part rather than the glottis in older patients.
- The neonatal airway is more prone to inspiratory collapse and obstruction due to a lack of posterior tracheal cartilage.
- Facial and head abnormalities, abnormal neck mobility, small mouth opening (small mandibles), and neck and airway masses may all interfere with visualization of the larynx, positioning, insertion of laryngoscope, tube placement, and ventilation both by mask and after intubation.

**Figure 1:**
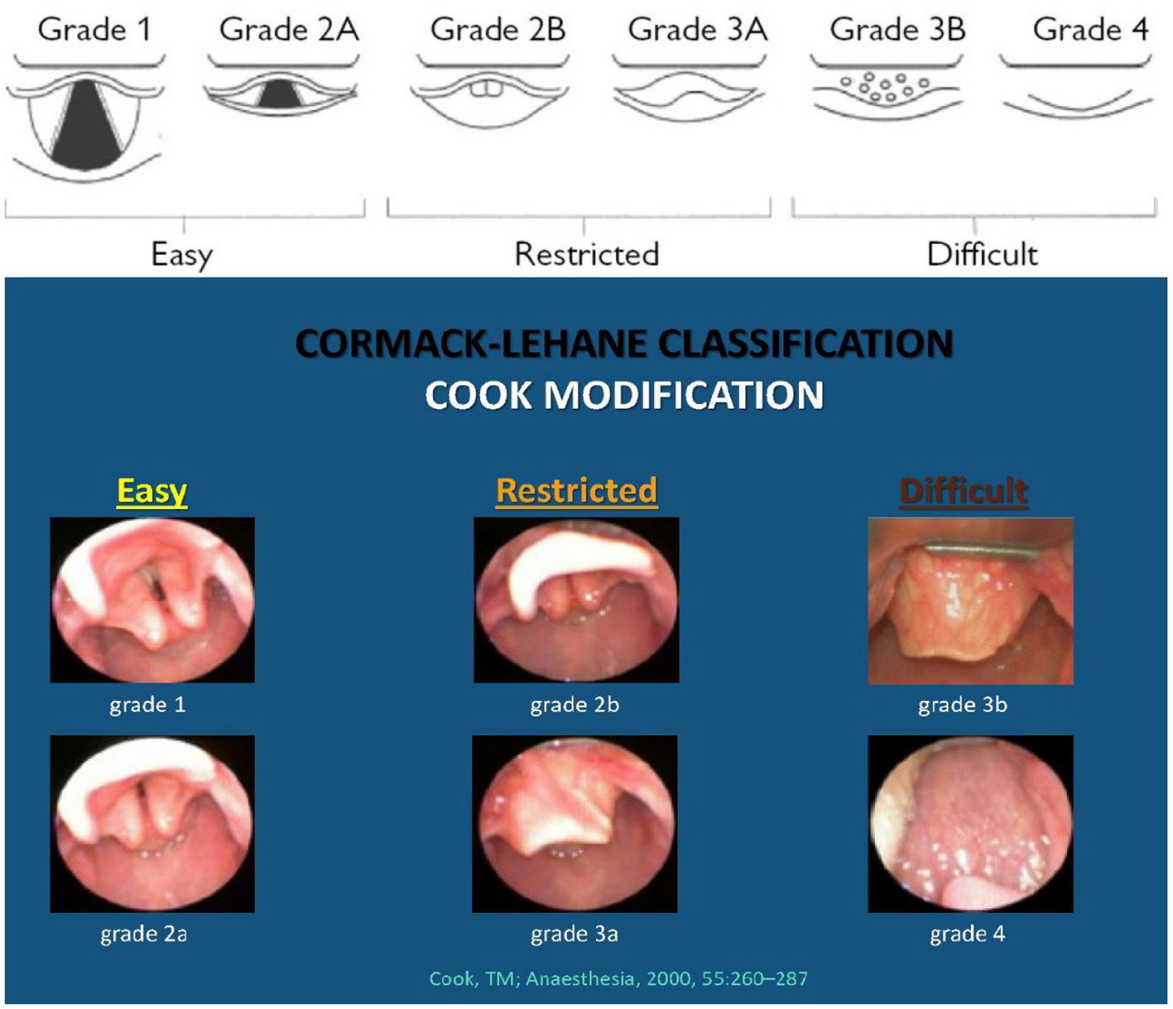
The modified Cook's practical classification of laryngeal view. “The view is *easy (E)* when the laryngeal inlet is visible. These views are suitable for intubation under direct vision. The view is *restricted (R)* when the posterior glottic structures (posterior commissure or arytenoid cartilages) are visible or the epiglottis is visible and can be lifted. These views are likely to benefit from indirect methods (e.g., gum elastic bougie). A *difficult (D)* view is present when the epiglottis cannot be lifted or when no laryngeal structures are visible. These views are likely to need advanced methods for intubation.” The figure and text are reproduced from “*A new practical classification of laryngeal view; Anesthesia, 2000, 55, 260–287*” with permission from Prof. T. M. Cook, MD, 21.01.2021, and RightsLink/John Wiley and Sons. The photographic part (with the blue background) minus photo “Grade 3b” (Grade 3b: Reprinted with permission from openairway.org.) along with other minor changes by author G. Berisha, MD, has been reproduced with permission from Carin A. Hagberg, MD, FASA, 08.10.2021.

Alignment of the trachea, the pharyngeal and laryngeal inlet must be achieved to avoid airway obstruction in the neonate during mask ventilation and to optimize the view of the laryngeal inlet during intubation. This can often be achieved by placing a towel roll under the shoulders to obtain a neutral position of the head and neck. “Grade of intubation views” is described in the Cormack-Lehane four-grade classification (8) and the modified Cook's classification (Figure 1) where “easy” views require no adjuncts; “restricted” views require a bougie; and “difficult” views require advanced techniques to intubate” (9). Neither of these classifications has been modified to apply to neonates.

### The Unexpected Difficult Airway in the Neonate

Whenever there are struggles with face mask ventilation, laryngoscopy, endotracheal intubation, and/or use of a supraglottic device “a difficult airway” situation arises (10, 11). In adult and pediatric intensive care, a ‘difficult intubation' involves three or more attempts at laryngoscopy by an experienced health care worker (HCW) (12). The incidence of difficult intubations in the adult intensive care unit (ICU) is 8–11% (13) and in the pediatric ICU 9% (14). The extent of intubation difficulties in the DR and NICU is unknown, but a review by Sawyer et al. (12) indicated a 14% NICU incidence. Difficult intubations were more common in small pre-mature neonates and highly linked to adverse events and severe oxygen desaturations (12).

Neonatal care in the first minutes of life focuses on lung aeration (15–17). Any difficulty encountered in opening and maintaining the airway may have dramatic cardiopulmonary consequences. A true difficult airway, e.g., airway malformation, may be rare in neonates (5). However, repeated instrumentation of the airway may result in bleeding, airway edema, and tissue trauma and a “can't ventilate, can't intubate” situation (5). Experience from anesthesiology is that algorithms, advance planning and routine practicing of a difficult airway approach optimize team responses to the technical and non-technical requirements of such situations (5). In neonatology, such advance plans may be lacking. We observed current approaches to advanced airway management in DR resuscitations in a Norwegian university hospital and reviewed the literature to provide recommendations about neonatal difficult airway management.

## Observations of DR Management of an Unexpected Difficult Airway

The Regional Committee for Medical and Health Research Ethics and the hospital institutional review board approved the project. Motion-activated video cameras with sound (Hikvision 2 megapixel IP camera, Hangzhou, China) were installed on all DR resuscitation cribs, focusing on the infant and the hands of the HCWs. All infants placed on a resuscitation crib August 2014-November 2016 were filmed and the videos were assessed for eligibility. The videos that contained positive pressure ventilation (PPV) were downloaded to a computer and transcribed. At the time of the video recordings, our NICU had no difficult airway algorithm or guideline. A research assistant with no practical neonatal resuscitation experience transcribed the videos using Interact software version 9 (Mangold Int GmbH, Arnstorf, Germany). A pediatrician (AMB) transcribed a selection of the recordings independently before the recordings were erased. There was minimal interrater bias. ALS, a consultant neonatologist, and GB, a consultant anesthesiologist, analyzed the transcripts quantitatively and qualitatively, and GB processed the data furthermore for statistical analyses.

This paper reports secondary analyses of some video transcripts that we previously reported in (1, 18, 19). The transcripts include information about profession/role of involved HCW, dialogue, provision of PPV and chest compressions (CC), and ETI attempts. The transcripts also include verbal assessment of PPV effectiveness, as well as visible chest rise and performance of ventilation corrective actions.

Twenty-three out of 314 (7.3%) eligible transcripts included ETI attempts. Table 1 presents characteristics of the infants that underwent intubation attempts and quality assessments of the attempts using the intubation subscale of the Neonatal Resuscitation Performance Evaluation (NRPE) score (20). In almost two thirds of the infants, three or more intubation attempts were required, and four infants spontaneously recovered after several failed attempts by multiple persons. Capnography was used in 7 (30%) of the patients (Table 1). Our NICU did not have laryngeal mask airway (LMA) or videolaryngoscopy (VL) available during the years of data collection. Stylets were available, but no pediatric or neonatal sized bougies. Use of a VL gives the benefit of direct observation of ETI. The benefit of direct observation also helps with troubleshooting e.g., if an infant does not improve despite successful intubation observed on VL, one should consider the possibility of a too deep ETT, a need to increase the inflation pressure, air leak, and thick viscous secretions needing deeper suction, rather than ending up in a vicious cycle of removing the ETT and reintubate.

**Table 1:** Characteristics of the infants (*n* = 23) that were intubated or underwent intubation attempts and the intubation attempts (*n* = 68).

**Characteristics of infants**	
Gestational age (weeks)	33 (28–40)
Weight (grams)	2,360 (1,070–3,590)
Female (number (%))	13 (57)
Apgar 1 min	2 (0–3)
Apgar 5 min	4 (2–5)
Apgar 10 min	7 (5–8)
Chest compression [number (%)]	12 (52)
Hypoxic-ischemic encephalopathy and therapeutic hypothermia [number (%)]	3 (13)
Died in the delivery room [number (%)]	2 (9)
**Characteristics of intubation attempts**	
Airway provider	
Pediatric resident	2
Pediatric specialist	6
Neonatologist	10
Anesthesiology resident	4
Anesthesiology specialist	1
Attempts	3 (2–6)
Duration (seconds)	44 (14–100)
Premedication [number (%)]	0 (0)
Suction [number (%)]	21 (91)
Positioning [number (%)]	23 (100)
Change endotracheal tube size [number (%)]	1 (4)
Capnography [number (%)]	7 (30)
Videolaryngoscopy [number (%)]	0 (0)
LMA [number (%)]	0 (0)
Oropharyngeal tube [number (%)]	3 (13)
Nasal intubation [number (%)]	11 (48)
Oral intubation [number (%)]	12 (52)
Intubation not successful [number (%)]^*^	4 (17)
Surgical airway [number (%)]	0 (0)
**Assessment of intubation attempts by the Neonatal Resuscitation Performance Evaluation (NRPE) score (20)**	
Appropriate decision based on clinical condition of infant e.g., prolonged ineffective PPV/bag mask, to facilitate CPR [number (%)]	20 (87)
Successful intubation in <3 attempts [number (%)]	8 (35)
Successful intubation in ≥3 attempts [number (%)]	11 (48)
Intubation not successful in ≥3 attempts and not achieved at all [number (%)]	4 (17)
One HCW tried intubation attempts [number (%)]	13 (57)
Two or more HCWs tried intubation attempts [number (%)]	7 (30)
Three or more HCWs tried intubation attempts [number (%)]	3 (13)
PPV recovery between attempts occurred [number (%)]	23 (100)
Position checked [number (%)]	21 (91)

* *Spontaneous recovery without intubation Data are presented as number (%) or median (interquartile range)*.

*LMA, Laryngeal Mask Airway; CPR, Cardiopulmonary resuscitation; PPV, Positive pressure ventilation; HCW, Health care worker*.

*Airway provider—Health care worker who performed the intubation and managed the airway, and number of infants intubated*.

Nasal and oral intubation was performed equally often. The mean (range) attempt duration was 44 (14–100) seconds, and the number of intubation attempts per HCW was 3 (2–6). Ten infants were successfully intubated by a consultant neonatologist and five by the anesthesia team. The remaining eight infants were intubated by a pediatric resident or consultant pediatrician. No pre-medication was given in any of the DR intubations (Table 1).

## Review of the Literature

We conducted a focused, but non-systematic search to find evidence of approaches to a difficult neonatal airway. The search was performed in MEDLINE, EMBASE, Pubmed Central and Web of science. The last search was performed 31.12.2020. We used a combination of key words and MeSH or EMTREE headings to describe “unexpected difficult airway in neonatal resuscitation,” “adverse events in neonatal tracheal intubation during resuscitation,” and “failed intubations in neonatal resuscitation.” Reviews, expert opinions, registry- and case studies were included, in addition to original studies.

### Mask Ventilation Difficulties

Face mask leak and airway obstruction are common during the first 2 min of PPV (21). Many reports suggest an increased risks of air leak with the widespread use of T-piece resuscitators in term resuscitation, and mask CPAP causing both brain and lung injuries i.e., intraventricular hematoma (22). Ineffective troubleshooting of suboptimal mask ventilation may occur partially due to a lack of knowledge of difficult airway management devices and skills for practical application (23).

### Nasal vs. Oral Intubation

In 2008, Jonathan Wyllie recommended that in an emergency, oral intubation should be the first choice as it decreases intubation time, is more likely to be successful on first attempt and is less traumatic (24). However, the choice between primary nasal and oral intubation remains mainly driven by institutional preference and HCW experience (25). Nasally placed tubes have been perceived to be more secure and comfortable for neonatal patients (25). However, a Cochrane review found no clear advantage of either nasal or oral intubation of newborn infants with respect to tissue trauma, infection, tube malposition/blockage/dislodgement, accidental extubation or re-intubation (26). Forty-one percent of neonatal patients presenting for cardiac surgery were nasally intubated in a retrospective study analyzing intubation route and perioperative outcomes (27). In this population, significantly less accidental and transesophageal echocardiogram related extubation occurred in infants that were nasally vs. orally intubated. There was no difference in airway complications between routes of intubation.

### Tracheal Tube Introducers

Tracheal tube introducers (TTI) also called gum elastic bougies are ‘difficult airway' devices that are also used as airway exchange introducers and to facilitate both intubation and extubation (28). Both TTIs and stylets have proven to be useful in the management of difficult airways in adults where early application is recommended (29). There are both straight and distal angled types TTIs for use down to endotracheal tube size 2.5–3.0–3.5 (30). Vygon's Bougie Boussignac has been used successfully in the intubation of infants with Pierre Robin Syndrome (31, 32) and TTIs and stylets combine well with videolaryngoscopy (33). Reports of complications with the use of bougies in neonates include pneumothoraxes and bronchial trauma (34) and controversy exist about under which circumstances, if any, a neonatal bougie should be used (34).

### Other Airway Adjuncts

Videolaryngoscope (VL) (35) and supraglottic airway devices (SAD) such as LMA (36) are increasingly used in neonates (37, 38) as companies develop equipment for use at lower gestations (39, 40). LMA is recommended in the 2020 European Resuscitation Council (ERC) guidelines as an adjunct in infants of 34 weeks gestation or more (17).

View of the glottis is improved by VL, which may also lower the risk of unfavorable events associated with neonatal ETI (5, 6). VLs are portable and can be used in both the DR and the NICU. VLs may be particularly useful in the management of an anticipated difficult airway situation e.g., airway malformations and anomalies (41). Trials have shown improved neonatal ETI success rates when trainees use VL instead of conventional laryngoscopy (12, 13) and VL during neonatal ETI has been independently associated with a reduction in unfavorable events (42). Overall, these findings suggest that VL may optimize the safety and success of neonatal ETI.

### Fiberoptic Intubation

The “gold standard” for difficult pediatric airway management is fiberoptic-guided ETI, i.e., fiberoptic bronchoscopy (FOB). However, there is a paucity of data on neonatal use of FOB during intubation and controlled extubation (43, 44). The technique of using a SAD as a conduit for FOB-guided intubation may reduce blurring of the vision caused by blood and secretions (45) and is recommended as an option to resolve the unanticipated difficult airway situation in children (29). Recent case reports and studies conclude that the technique is an effective and safe option in the anticipated and unanticipated difficult airway in neonates and infants (43, 44).

### Pre-medication

If time allows, systemic pre-medication should be used to optimize the chance of achieving ETI at the first attempt. However, sick neonates at delivery, in the NICU, or in the operating room may lack physiological reserve, pre-disposing to rapid compromise when innate respiratory effort is lost (4, 46, 47). Especially the use of muscle relaxants may cause a situation of “point of no return.” In the case of an expected difficult airway, e.g., short neck or hypotonia, an alternative approach to relaxation may be topical lidocaine at the laryngeal inlet (48–50). Topicalization of the airway involves applying a local anesthetic over an area of mucosa to achieve regional neural blockade. Topical lidocaine reduces airway reflexes, swallowing reflex and cough by blocking sensory receptors. Airway instrumentation and intubation can be performed 1.5–2 min after lidocaine topicalization, e.g., with customized spray devices (50). The recommended neonatal dose is 3–4 mg/kg (50). Lidocaine topicalization can shorten the neonatal intubation time whilst preserving blood pressure, heart rate and oxygen saturation (49).

Nasal drugs for procedural sedation and analgesia in neonates are increasing (51–55) and may be used as pre-medication before intubation of newborns. Milési et al. compared intranasal midazolam and ketamine for neonatal intubation in pre-term neonates and found that the initial respiratory and hemodynamic tolerance of the two drugs were comparable despite 2–4 mg/kg nasal ketamine being less efficient than nasal midazolam in rapidly achieving adequate sedation for intubation (54). Also of note, despite relatively little work reported on its use, when having ventilation and intubation difficulties, intralingual or submental suxamethonium offers a possibility to resolve the problem (56).

### Emergency Surgical Airway/Front of Neck Access (FONA)

Establishing a front of neck airway in infants <2 kg or <36 weeks post-menstrual age can be difficult due to the neonatal airway anatomy, especially the short neck and relatively thick anterior neck tissues (57, 58). Under general anesthesia, an elective surgical airway (tracheostomy) can be established in neonates weighing <2 kg. Suboptimal conditions and the added stress and time pressure in an unexpected difficult airway situation, are likely to reduce the chances of success even in trained hands. In adults and older children, the neck anatomy allows for needle cricothyroidotomy as a potential rescue technique in a “can't intubate/can't ventilate” scenario. An attempt to perform needle cricothyroidotomy as the first FONA option is recommended in children in the absence of an ENT specialist (59, 60). However, unfortunately, this procedure has been shown to have a failure rate of 65% in adults (61). In neonates, cricothyroidotomy by scalpel or needle is not possible because of the small sized cricothyroid membrane and neonatal subglottis (62, 63). Establishing effective respiration (oxygenation and ventilation) rapidly *while avoiding damage to the anatomical structures* is the goal of a neonatal surgical airway. Therefore, FONA is not recommended for the management of a difficult neonatal airway in centers without pediatric ear, nose, and throat (ENT) services (5). A “can't intubate/can't ventilate” scenario is rare and frightening. Training for such events is difficult as simulator and airway models only partially resemble the neonatal airway anatomy. Regular training likely decreases HCWs reluctance to establish a surgical airway. Both technical and non-technical skills needed for performing a surgical airway and managing a neonatal “can't intubate/can't ventilate” scenario should be practiced and trained.

### Policies and Procedures

Skilled airway management is required even for anatomically normal neonatal airways (64). In expected and unexpected difficult intubation in children, patients <10 kg are more prone to complications than larger patients (65). This is because of rapid desaturation due to a lower functional residual capacity and higher oxygen consumption, as well as lower success rates of advanced airway rescue techniques (25, 66). Therefore, any airway management procedure should anticipate the possible failure of and plan one step ahead. Policies for managing neonatal intubation depend on the clinical setting in which the care is being given (6). In UK NICUs a difficult airway approach or plan is not universally present (67). This is expressed as variations in the allowed or expected number/quality/duration of intubation attempts by a single HCW (68, 69). Essentially, the escalation pathway if the most senior neonatologist/pediatrician is unable to stabilize the neonatal airway including involving ENT surgeons (68), pediatric respiratory specialists or consultant anesthesiologist with airway expertise, should be formalized in policies and procedures tailored to the individual context.

### Simulation and Practice

The ERC Newborn Life Support guidelines (2) emphasize airway management as the fundamental component of neonatal stabilization and/or resuscitation at birth. Awareness has increased regarding the importance of human factors, including crew resource management (CRM) and systems optimization for successful preparation in dealing with the difficult airway situation (29). Simulation training to acquire and maintain skills, and practicing non-technical skills including communication, CRM and leadership are recommended (70, 71). Implementation is also a major consideration being more than just simulation and practice and must be done thoroughly so that all involved doctors can learn the designated steps in the suggested algorithm. Communication according to CRM principles and the method of “closed loop” communication together with team members having defined roles and function, is current practice at AUH.

## Discussion

In older children, a difficult airway situation arises more frequently at intubation for surgery, i.e., in a controlled and well-equipped environment with an airway expert present. In the neonate, including in the DR, the need for intubation is more often unexpected. The rate of three or more intubation attempts in our DR observations was 65%. Four out of twenty-three patients (17%) were not intubated despite multiple attempts at securing the airway, and in 11 (48%) patients, three or more attempts were needed to achieve successful ETI. In a multicenter NICU intubation registry (12), a difficult airway situation, defined as the need for three or more attempts, occurred in 14% of patients, considerably less than what we observed in our hospital.

Guidelines for difficult airway management in older children and adults include the Difficult Airway Society 2015 guidelines for adults and children (29), and recommendations from airway focus groups and societies (10, 72–74). Similar guidelines for children also exist (59, 60, 75, 76) and these are used daily in countries all over the world. While a recent guideline from the British Pediatric Society exists, and the Scottish NHS and Advanced Resuscitation of the Newborn Infant (ARNI) airway management algorithm was recently revised, there is no Norwegian national guideline for managing the unanticipated difficult airway in the DR and NICU. After our DR observations, still no algorithm exists in our unit, while a Karl Storz CMAC videolaryngoscope has been purchased. However, there is no guideline to suggest in which situations the VL should be used.

**Figure 2:**
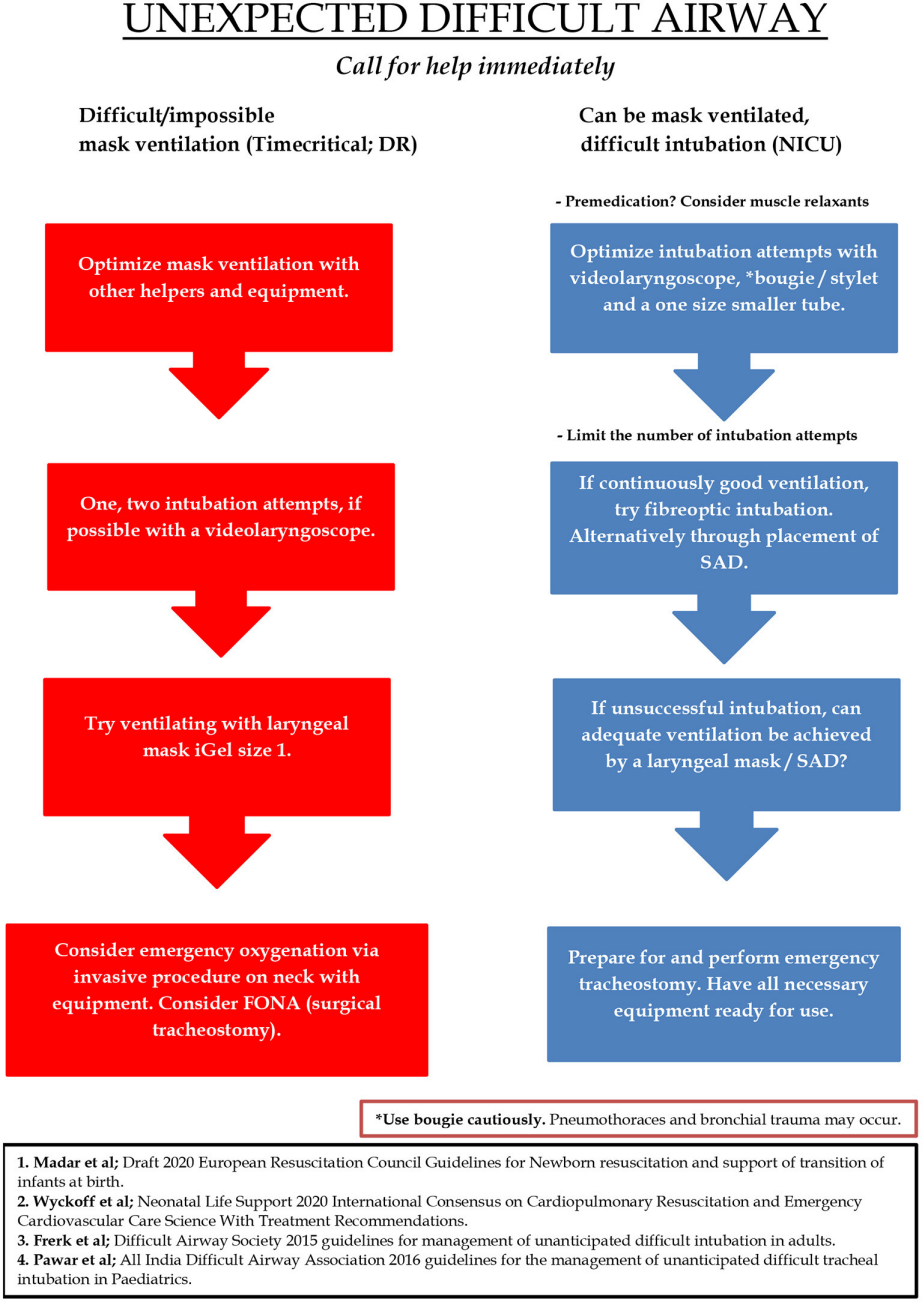
Proposed algorithm for the management of the neonatal unanticipated difficult airway. The red column describes the situation where mask ventilation is difficult and/or impossible to achieve despite measures including the insertion of an oropharyngeal or nasopharyngeal tube, and two-person mask ventilation. The red column is the most time critical. The blue column is the situation where mask ventilation is possible, but the newborn is difficult to intubate, e.g., a semi-vigorous baby where positive pressure ventilation is considered ineffective or prolonged. In this situation there is usually time to reevaluate the initial management plan and reposition the head of the newborn for another intubation attempt with a videolaryngoscope. DR, Delivery Room; FONA, Front of neck access; NICU, Neonatal intensive care unit; SAD, Supraglottic airway device.

Based on our literature review, stylets and bougies, in combination with videolaryngoscopy may potentially have improved the success rate of ETI in the difficult airway situations in our DR. However, use of neonatal bougies remains controversial. An LMA could have been used if ETI was unsuccessful. Based on a remarkably high incidence of a difficult airway situation in the DR and a literature review, we propose a simple management-algorithm for the neonatal unanticipated difficult airway (Figure 2).

The main goals of the algorithm are to minimize intubation attempts, quickly achieve adequate oxygenation and ventilation, and establish a definite airway by either the insertion of a LMA or successful ETI. Calling for help early and making sure that the most competent airway provider is present is crucial. Importantly, to optimize intubation conditions, pre-medication and muscle relaxants should be given whenever time allows it. FONA is technically difficult in neonates and should only be performed by ENT surgeons.

The algorithm is simple in order to be intelligible in a time-critical and stressful situation. But also, for the suggested algorithm to inform and improve practice it should be learned and trained in simulations and under supervision in real-life situations. A difficult airway and intubation can occur anywhere and at any time of the day or night, and the algorithm is not site-specific for use only in the DR or NICU. The red and blue columns can also be a consequence of one another. For example, if mask ventilation is possible while intubation is difficult, the administration of analgesics and muscle relaxants may result in a “no longer able to ventilate” situation while intubation is still difficult. In such a situation, measures to achieve successful ETI must be performed faster while the situation becomes more stressful.

Limitations of our empirical data are present. The raw data video recordings were not available as all videos were, for confidentiality reasons, erased after initial review and transcription. This is the main reason why more details of the intubation attempts are lacking, e.g., the causes for the multiple failed intubation attempts. Extremely pre-term neonates from 22 to 27 + 6 weeks gestation are more likely to present major difficulties during mask ventilation and intubation due to their small size and more unstable condition in general, but are underrepresented in our material.

In conclusion, based on our observations of DR resuscitations and a review of the literature, we propose a “neonatal difficult airway algorithm” that includes limiting individual provider ETI attempts and use of airway adjuncts. Our recommendations also include practice and preparations for the non-technical requirements of dealing with the stress of an unexpected difficult airway.

## Data Availability Statement

The raw data supporting the conclusions of this article will be made available by the authors, without undue reservation.

## Ethics Statement

The studies involving human participants were reviewed and approved by the Regional Committee for Medical and Health Research Ethics South East and the institutional review board at Akershus University Hospital. The institutional review board approved presumed consent from the parents (reference 14-032).

## Author Contributions

GB and AS conceptualized and designed the study, performed data analysis and interpretation, and drafted the initial version of the manuscript. AB performed the data collection, structured the data, and was involved in the data analysis and interpretation. EB and AR were involved in the data analysis and interpretation. All authors participated in critical revision of the manuscript for important intellectual content and approved the final manuscript as submitted and agree to be accountable for all aspects of the work.

## Funding

This research was supported by the Laerdal Foundation for Acute Medicine, Stavanger, Norway.

## Conflict of Interest

The authors declare that the research was conducted in the absence of any commercial or financial relationships that could be construed as a potential conflict of interest.

## Publisher's Note

All claims expressed in this article are solely those of the authors and do not necessarily represent those of their affiliated organizations, or those of the publisher, the editors and the reviewers. Any product that may be evaluated in this article, or claim that may be made by its manufacturer, is not guaranteed or endorsed by the publisher.
