# Corrigendum: Management of the unexpected difficult airway in neonatal resuscitation

**DOI:** 10.3389/fped.2022.1124050

**Published:** 2023-01-25

**Authors:** Gazmend Berisha, Anne Marthe Boldingh, Elin Wahl Blakstad, Arild Erlend Rønnestad, Anne Lee Solevåg

**Affiliations:** ^1^ Department of Paediatric and Adolescent Medicine, Akershus University Hospital, Lørenskog, Norway; ^2^ Institute of Clinical Medicine, Faculty of Medicine, University of Oslo, Oslo, Norway; ^3^ Department of Neonatal Intensive Care, Division of Paediatric and Adolescent Medicine, Oslo University Hospital, Rikshospitalet, Oslo, Norway

**Keywords:** algorithm, difficult airway, endotracheal intubation, newborn, resuscitation

A Corrigendum on Management of the unexpected difficult airway in neonatal resuscitation By Berisha G, Boldingh AM, Blakstad EW, Rønnestad AE and Solevåg AL. (2021) Front Pediatr. 9:699159. doi: 10.3389/fped.2021.699159

In the published article, there was an error regarding the affiliation(s) for Gazmend Berisha. As well as having affiliation 1, he should also have Institute of Clinical Medicine, Faculty of Medicine, University of Oslo, Oslo, Norway.

In the published article, there was an error regarding the affiliation(s) for Arild Rønnestad. As well as having affiliation 2, he should also have Institute of Clinical Medicine, Faculty of Medicine, University of Oslo, Oslo, Norway.

In the published article, there was an error in the Ethics statement.

A correction has been made to **[Ethics Statement]**. The statement previously stated:

“[The studies involving human participants were reviewed and approved by the Norwegian Health Directorate (HDIR) and Klinisk Etisk komite (REK) at Akershus University Hospital. Written informed consent to participate in this study was provided by the participants’ legal guardian/next of kin. Written informed consent was obtained from the minor(s)' legal guardian/next of kin for the publication of any potentially identifiable images or data included in this article.]”

The corrected sentence appears below:

“[The studies involving human participants were reviewed and approved by the Regional Committee for Medical and Health Research Ethics South East and the institutional review board at Akershus University Hospital. The institutional review board approved presumed consent from the parents (reference 14-032]”

The authors apologize for these errors and state that they do not change the scientific conclusions of the article in any way. The original article has been updated.

